# 3D Self‐Architectured Steam Electrode Enabled Efficient and Durable Hydrogen Production in a Proton‐Conducting Solid Oxide Electrolysis Cell at Temperatures Lower Than 600 °C

**DOI:** 10.1002/advs.201800360

**Published:** 2018-08-31

**Authors:** Wei Wu, Hanping Ding, Yunya Zhang, Yong Ding, Prashant Katiyar, Prasun K. Majumdar, Ting He, Dong Ding

**Affiliations:** ^1^ Energy and Environmental Science and Technology Idaho National Laboratory Idaho Falls ID 83415 USA; ^2^ School of Materials Science and Engineering Georgia Institute of Technology Atlanta GA 30332 USA; ^3^ Department of Mechanical Engineering University of South Carolina Columbia SC 29208 USA

**Keywords:** 3D electrodes, interfaces, proton‐conducting oxide, solid oxide electrolysis cells, water splitting

## Abstract

Hydrogen production via water electrolysis using solid oxide electrolysis cells (SOECs) has attracted considerable attention because of its favorable thermodynamics and kinetics. It is considered as the most efficient and low‐cost option for hydrogen production from renewable energies. By using proton‐conducting electrolyte (H‐SOECs), the operating temperature can be reduced from beyond 800 to 600 °C or even lower due to its higher conductivity and lower activation energy. Technical barriers associated with the conventional oxygen‐ion conducting SOECs (O‐SOECs), that is, hydrogen separation and electrode instability that is primarily due to the Ni oxidation at high steam concentration and delamination associated with oxygen evolution, can be remarkably mitigated. Here, a self‐architectured ultraporous (SAUP) 3D steam electrode is developed for efficient H‐SOECs below 600 °C. At 600 °C, the electrolysis current density reaches 2.02 A cm^−2^ at 1.6 V. Instead of fast degradation in most O‐SOECs, performance enhancement is observed during electrolysis at an applied voltage of 1.6 V at 500 °C for over 75 h, attributed to the “bridging” effect originating from reorganization of the steam electrode. The H‐SOEC with SAUP steam electrode demonstrates excellent performance, promising a new prospective for next‐generation steam electrolysis at reduced temperatures.

## 1 Introduction

Renewable and sustainable energies have gained intensive attention worldwide during the past decades, as the consumption of fossil fuels causes serious environmental issues. Hydrogen, one of the most abundant elements in the earth, is regarded as the cleanest fuel that has great potential to replace the carbon‐based fuels.^1^ Consequently, the high‐efficient production of hydrogen becomes crucial to the hydrogen economy. At present, most hydrogen is produced from steam reforming of fossil fuels (steam methane reforming, SMR),^2^ which is a mature technique due to its operational reliability and low cost of methane since the shale gas revolution. However, it has to be acknowledged that SMR is neither sustainable nor eco‐friendly, since the fossil fuels are not renewable and the exhausts (e.g., CO_2_ and SO_2_) are threats to the environment. Most importantly, SMR has low‐cost scaling factor of a conventional reformer, which is a relatively expensive option for distributed or small‐scale applications, especially when it becomes increasingly important to combine other renewable energy technologies together. Consequently, steam electrolysis is a viable alternative to SMR in specific applications owing to its very high‐cost scaling factors and clean products. The overall reaction follows the equation

(1) $$H_{2}O\to H_{2}+\frac{1}{2}O_{2}$$

Among all the steam electrolysis techniques, solid oxide electrolysis cells (SOECs) at elevated temperatures provide several benefits over low‐temperature electrolysis technologies, including the reduced electricity demand, fast electrode kinetics, and less expensive materials (e.g., Ni rather than Pt) can be used as electrode catalysts.^3^

SOEC is the reversible operation of solid oxide fuel cell (SOFC), and can be divided into two categories due to the different types of the electrolyte: the oxygen ion‐conducting SOECs (O‐SOECs) and the proton‐conducting SOECs (P‐SOECs). The conventional SOECs typically use oxygen ion‐conducting yttria‐stabilized zirconia as electrolyte materials,^4^ which have to be operated at high temperatures to ensure reasonable cell performance due to the insufficient ionic conductivity at low temperatures. However, running electrochemical cells at high operating temperatures has obvious problems such as fast degradation,^5^ sealing difficulty,^6^ poor start‐up and thermal cycling,^7^ etc. Moreover, electrode will delaminate at high current density, resulting from extreme high oxygen partial pressure at the electrolyte–electrode interface.^8^ P‐SOECs, on the other hand, are able to operate at lower temperatures due to the higher ionic conductivity of proton‐conducting electrolytes compared with that of oxygen ion‐conducting electrolyte at reduced temperatures.^9^ In addition, P‐SOECs produce pure and dry H_2_ only at hydrogen electrode side since the proton‐conducting electrolyte is nonpermeable to both oxide ions and molecular gases at low temperatures.^3^ As nickel is widely used in the hydrogen electrode for SOECs,[[qv: 4b,10]] P‐SOECs can prevent the Ni oxidation at high steam concentration, which is one of the reasons for performance degradation in O‐SOECs.^11^ Therefore, for these advantages toward traditional O‐SOEC, P‐SOECs have gained much attention in recent years.^12^

A series of perovskite proton‐conducting oxides, such as doped BaCeO_3_,^13^ SrCeO_3_,^14^ and BaZrO_3_,^15^ have been applied as electrolyte materials in SOECs at temperature range of 600–800 °C. Among which, Y‐doped solid solution of BaZrO_3_ and BaCeO_3_ (BZCY) seems the best choice that compromises both high conductivity and good chemical stability.^9^, ^16^ In order to further optimize performance, modification of BZCY materials has gained much attention.^17^ Liu group developed a mixed proton conductor, BaZr_0.1_Ce_0.7_Y_0.2−_ *_x_*Yb*_x_*O_3−_ *_δ_* (BZCYYb) for SOFCs, which exhibits high ionic conductivity and hydrogen permeability at intermediate temperatures, as well as enhanced water adsorption.^18^ Very recently, Kim et al. reported a “hybrid” SOEC using BZCYYb and NdBa_0.5_Sr_0.5_Co_1.5_Fe_0.5_O_5+_ *_δ_* (NBSCF) as electrolyte and steam electrode, respectively.^19^ This is due to the mixed protonic and oxygen ionic conduction in the electrolyte at elevated temperatures (>600 °C). At the temperature below 600 °C, it has been confirmed that this material family exhibited a proton transference number >90%.^20^ This suggested that BZCYYb could possess a pure proton conduction at reduced temperatures. Unfortunately, the applications of BZCYYb in SOECs still lack of investigation, especially at the temperatures lower than 600 °C.

Apart from the recent progress in development of proton‐conducting electrolytes for SOECs, the design of appropriate steam electrode for good cell performance still remains elusive, especially at low temperatures.^21^ A good steam electrode for SOECs should have sufficient pathways for both electron/proton and gas diffusion, as well as high surface area for better catalytic reaction activity. Therefore, both morphology and porosity of the electrode play the key roles for electrochemical performance in the electrode. In the former case, Liu et al.^22^ developed an electrode supported SOEC with dual‐layer structure steam electrode: a thin sponge‐like pore layer is supported on a thick finger‐like macrovoid layer. The finger‐like macrovoid electrode provided vertical aligned channels for gas transport and thus enhanced cell performance. However, the electrode porosity is relatively small (≈28%), suggesting large space to improve porosity. On the other hand, Suzuki et al. investigated the correlation between the electrode porosity and cell performance. They concluded that higher linear fuel velocity led to better cell performance, as validated in those cells with higher porosity in the electrode.^23^ Because the size of water molecule (≈275 pm) is larger than that of O_2_ molecule (≈150 pm), the steam electrode in SOECs requires more porosity than cathode in SOFCs for mass transfer. Consequently, the concept of ultrahigh porous structure has been developed and proved to be effective to improve the electrode performance.^24^ For examples, Chen et al. reported a 3D fibrous porous cathode for intermediate SOFCs, exhibiting dramatically improved cell performance at 550 °C.^25^ The oxygen reduction reaction was greatly enhanced by hollow fiber networks with calculated high porosity and straight path for electrode reactions. In order to simplify the fabrication and eliminate the use of high voltage, Dong et al. developed a template‐derived method to fabricate highly porous, interwoven fibrous Sm_0.5_Sr_0.5_CoO_3_ (SSC) cathode for SOFCs.^26^ The maximum power density of the cell with the templated porous cathode increased by 44.5 and 29.8% at 600 and 500 °C, respectively, comparing with that made with combusted SSC. This work indicated the effect of porosity in the fibrous electrode on electrode kinetics by enlarged triple‐phase boundary and enhanced mass transfer. However, the fabrication processes of such highly porous cathode were complicated, and it was challenging to incorporate them into the full cells with the structure integrity. In addition, its strength and flexibility are supposed to be poor, resulting in difficulty of mass production. Therefore, it is greatly desirable to develop highly porous electrode with aligned microstructure that ensures adequate mass transfer pathways and sufficient mechanical strength in SOECs.

Besides the electrode microstructure consideration, the electrical properties of steam electrode material are also crucial to the SOEC performance. According to Grimaud et al., some well‐known oxygen ion‐conducting oxides (e.g., Ba_0.5_Sr_0.5_Co_0.8_Fe_0.2_O_3−_ *_δ_*, BSCF and PrBaCo_2_O_5+_ *_δ_*, PBCO) presented certain proton conduction in wet atmosphere, which could be beneficial for the steam electrode reaction under electrolysis conditions.^27^ Those oxides that possess electronic, oxygen ion and proton conductivities are labeled as triple‐conducting oxides (TCOs). Choi et al. reported a TCO material, PrBa_0.5_Sr_0.5_Co_2−_ *_x_*Fe*_x_*O_5+_ *_δ_* (PBSCF), which was successfully applied as SOFC cathode with a peak power density ≈2.2 W cm^−2^ at 600 °C.^28^ In Kim's work, they employed triple conducting NdBa_0.5_Sr_0.5_Co_1.5_Fe_0.5_O_5+_ *_δ_* (NBSCF) as steam electrode and demonstrated excellent SOEC performance. However, the Faraday efficiency in this “hybrid” SOEC could be expectedly low owing to the aggravated current leakage of BZCYYb electrolyte at higher temperatures.^20^, ^29^

Inspired by the merits of highly porous electrode and outstanding performance of PBSCF, we developed a novel self‐architectured ultraporous (SAUP) 3D steam electrode consisting of hollow PBSCF fibers for water splitting reaction in this study. The fibers exhibited excellent strength and flexibility upon commercially available coupons. The SOEC consisting of a SUAP PBSCF textile steam electrode, BZCYYb electrolyte, and Ni‐BZCYYb hydrogen electrode has demonstrated excellent efficiency and durability in steam electrolysis below 600 °C. The electrochemical tests were performed in 5%H_2_‐95%Ar as the hydrogen electrode purge gas and 12%H_2_O‐88%O_2_ as the steam electrode inlet gas.

## 2 Results and Discussion

### 2.1 Characterization of SAUP 3D Steam Electrode

**Figure 1:**
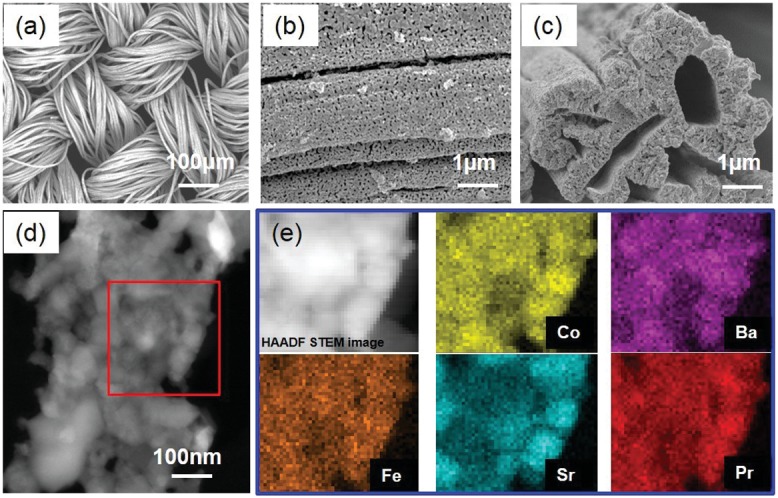
3D PBSCF anode framework: a) SEM image of a sintered 3D PBSCF framework surface; b) a closer view of the wall of fibers, showing uniformly distributed open pores; c) cross‐sectional image of hollow fibers; d) HAADF STEM image of a piece from fiber wall; e) EELS mapping from the red square area marked in (c).

**Figure** **1**a shows a scanning electron microscopy (SEM) image of a calcined 3D PBSCF framework. The textile‐like structure that consists of bundles of fibers provides the framework with sufficient mechanical strength and flexibility to be processed and integrated onto the final cell compared to those randomly aligned nanofiber electrodes,^25^ as well as adequate porosity to ensure fast mass transfer. Nanosized pores are evenly distributed throughout the wall of each single fiber, as shown in Figure 1b, which increase the specific surface area of fibers. High‐magnitude cross‐sectional image of fibers is shown in Figure 1c. The fibers are hollow with an average inner diameter of 1–2 µm, allowing water molecules to further go inside the electrode fibers at operating temperatures. The detailed features of the hollow fibers are further revealed by scanning transmission electron microscopy (STEM). Figure 1d is the high‐angle annular dark‐field (HAADF) STEM image of one thin piece from the hollow fiber. Figure 1e is the electron energy‐loss spectroscopy (EELS) mapping from the area marked in Figure 1d, showing a relatively uniform distribution of all Pr, Ba, Sr, Co, Fe elements. The phase purity of this ceramic textile framework was also examined with X‐ray diffraction (XRD). The standard phases of PBSCF were confirmed (see Figure S3, Supporting Information), indicating that the fibers were well synthesized with no secondary phase.

**Figure 2:**
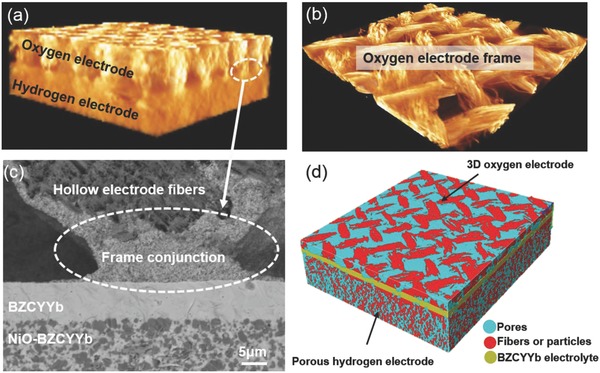
a) 3D X‐ray microscopic image for the cell consisting of hydrogen electrode (bottom layer), electrolyte (invisible) and 3D steam electrode (top layer). b) The bulk electrode frame with hierarchical gaps, which would facilitate fast mass transfer; c) SEM image for the cross section of the cell shows the contact area between steam electrode frame and electrolyte and d) reconstructed 3D image for the cell with dimension of 0.4 × 0.4 × 0.1 mm^3^. Three layers of cell components, that is, fibrous PBSCF electrode with a porosity of 57.7%, dense BZCYYb electrolyte, and sponge‐like pore Ni‐BZCYYb hydrogen electrode support, are obviously distinguished.

The novel cell microstructure, especially the interface between 3D steam electrode and BZCYYb electrolyte, was also characterized by X‐ray microscopy, reconstructed 3D, and also scanning electron microscope, as shown in **Figure** **2**. The images were taken from the cell before electrochemical test, which typically demonstrated the initial morphology of cell components. Figure 2a shows the 3D X‐ray microscopic image of 3D steam electrode (top), electrolyte (middle), and hydrogen electrode (bottom) tri‐layer with a dimension of 0.6 × 0.6 × 0.2 mm^3^. The BZCYYb electrolyte layer was hard to distinguish due to the limited thickness (10 µm) compared with the overall large scale. Interface contacts between 3D electrode and electrolyte were clearly observed, which is critical to electrode bonding as well as charge transfer. Moreover, the bulk fiber frame presents no crack or disconnection after application on the electrolyte, as shown in Figure 2b. The cross‐sectional SEM image showing the interface contact between electrodes and electrolyte before electrolysis is presented in Figure 2c. As shown in the figure, both the 3D steam electrode and the porous hydrogen electrode are well adhered to the dense BZCYYb electrolyte, forming delicate interfaces of electrolyte and electrode. Clear interface contacts between 3D steam electrode and BZCYYb electrolyte were observed in both X‐ray microscopy (Figure 2a) and SEM (Figure 2c). These solid contacts consist of PBSCF particles from PVB/PBSCF suspension in bonding process, which moved through the frame gaps to the interface under the force of gravity to form a pier‐like conjunction. A microstructure comparison of 3D and conventional PBSCF steam electrodes is demonstrated in Figure S4 (Supporting Information). To further study the 3D steam electrode structure porosity, a 3D microstructure has been reconstructed by directly stacking the raw X‐ray images along the *x*‐axis in sequence. The reconstructed image is shown in Figure 2d with a dimension of 0.4 × 0.4 × 0.1 mm^3^. The porosity of bulk steam electrode was 57.7%, which was obtained as the number fraction of pore pixels in the 2D slice. The porosity is much higher than those hierarchically oriented macroporous electrode reported by Chen et al.[[qv: 24b]] The ultralarge porosity could benefit to the fast steam transfer within the steam electrode and subsequently enhance the steam electrolysis performance.

### 2.2 Electrochemical Performance

**Figure 3:**
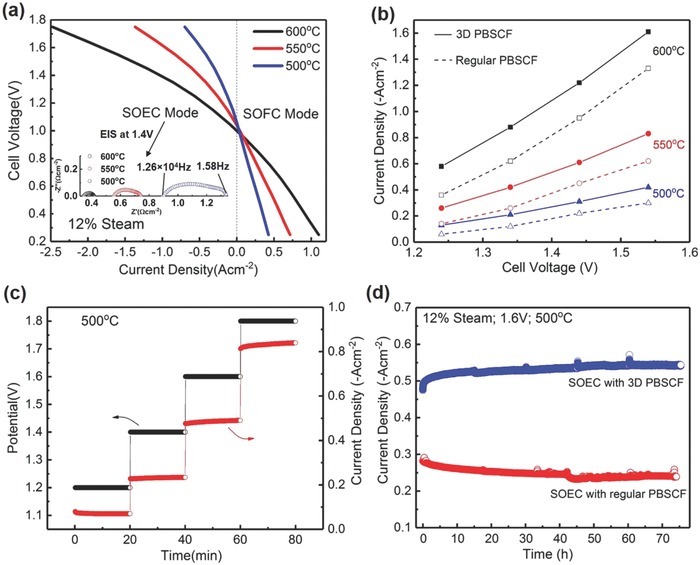
a) *I*–*V* curves of solid oxide cell measured in SOEC and SOFC mode at various temperatures; b) electrolysis performance enhanced by SAUP 3D PBSCF steam electrode when compared with that of conventional PBSCF electrode at different temperatures; c) short‐term electrolysis at different applied voltages at 500 °C and d) durability of SOECs with 3D PBSCF steam electrode (blue) and conventional PBSCF steam electrode (red) under applied voltage of 1.6 V at 500 °C. The performance of SOEC with conventional PBSCF degrades with time while that of SOEC with 3D PBSCF shows constant activation.

The electrochemical test was performed at a temperature range of 500–600 °C with a steam partial pressure of 12%, which was obtained by setting the temperature of inlet carrying gas bubbler to 50 °C. The voltage–current characteristics of the solid oxide cell in both SOFC and SOEC mode were recorded when pure H_2_ was introduced into the hydrogen electrode, as shown in **Figure** **3**a. The positive current density refers to fuel cell operation while the negative current density refers to electrolysis cell operation. Cell potential values at zero current density correspond to the open‐circuit voltages (OCV). The OCVs of the cell are 0.99, 1.02, and 1.04 V at 600, 550, and 500 °C, respectively, which are slightly lower than the OCV values at 3% steam partial pressure reported previously.[[qv: 12a]] The OCV difference came from the lower oxygen partial pressure when the steam ratio increased from 3 to 12%. In SOFC mode, the slope of *I*–*V* curve increases with decrease of operating temperature, which indicates that the Ohmic resistance of cell increases. The area specific resistances (ASRs) of the cell in SOFC mode can be calculated from the slope of the *I*–*V* curves with the section of potential between 0.3 and 0.8 V where the *I*–*V* curve presented linearly. The ASRs of the cell in SOFC mode are ≈0.72, 1.13, and 1.86 Ω cm^2^ at 600, 550, and 500 °C, respectively. When the SOFC was operated at 0.7 V, the current densities are 0.52, 0.32, and 0.13 A cm^−2^ with power densities of 0.36, 0.22, and 0.09 W cm^−2^ at 600, 550, and 500 °C, respectively. The performance in SOFC mode was reasonable and even 1.6 times of the peak power density of the Ni‐BZCYYb anode supported fuel cell with BaCo_0.4_Fe_0.4_Zr_0.2_O_3−_ *_δ_* cathode.^30^ While in SOEC mode, the steam electrolysis performance demonstrated the same trend as the performance change in SOFC mode. When the cell was operated at a potential of 1.6 V, the electrolysis current densities are −2.02, −1.08, and −0.54 A cm^−2^ at 600, 550, and 500 °C, respectively. The steam electrolysis performance in this work at 500 °C is even better than that of P‐SOEC at 700 °C reported by Gan et al.,^31^ as well as the electrolysis performance of a GDC‐based O‐SOEC reported by Heidari et al.^32^ According to the inserted electrochemical impedance spectroscopy (EIS) results in Figure 3a, the Ohmic ASRs at 1.4 V are 0.34, 0.55, and 0.9 Ω cm^2^ for 600, 550, and 500 °C, respectively, which are significantly lower than that obtained in SOFC mode with similar cell configuration.^33^ It can be interpreted by the fact that the presence of water leads to the formation of more protonic defects in oxygen‐deficient perovskite electrolyte^34^ and thus increases its conductivity.^35^

Figure 3b compares the electrolysis performances between SOECs with conventional and SAUP 3D PBSCF steam electrode at different temperatures. At each operating temperature, the current densities of SOEC with 3D steam electrode at different applied voltages were much higher than those of cell with conventional PBSCF steam electrode, which indicated an obvious improvement of electrolysis performance with the application of 3D steam electrode. Figure 3c presents the electrolysis step test at different applied voltages of 1.2, 1.4, 1.6, and 1.8 V at 500 °C. A current density of ≈0.5 A cm^−2^ was observed at 1.6 V, which is in good accordance with that in Figure 3a. At each stage, the electrolysis current is stable at low applied voltage and keeps increasing at higher one, presenting an “activation” process. This is evidenced by the observation that the current density stayed the same at 1.4 V while the cell was changed from −0.79 to −0.83 A cm^−2^ at 1.8 V. The relevant Faraday efficiency at each applied voltage was calculated and listed in **Table** **1**. According to the Faraday's law, the theoretical hydrogen production rate (100% Faraday efficiency) converted from the electronic balance and equivalent hydrogen production flow rate can be defined as follows

(2) $$\dot{V}\;=\;\frac{1}{2F}\times V_{m}\times t$$

where $\dot{V}$ is the theoretical hydrogen production flow rate (standard cubic centimeter per minute, sccm), *I* is the input current (A), 2 is the number of electrons involved in the steam electrolysis reaction, *F* is the Faraday constant, and *V*_m_ is the molar volume of a gas (22 400 mL mol^−1^). The experimental hydrogen production flow rate *V*_e_ is obtained by analyzing the gas composition of hydrogen electrode exhaust using a gas chromatography (GC). Therefore, the Faraday efficiency η could be defined as follows

(3) $$\eta \;=\;\frac{V_{e}}{\dot{V}}$$

**Table 1:** Faraday efficiency at different applied voltages at 500 °C

Applied voltage [V]	1.2	1.4	1.6	1.8
Current density [A cm^−2^]	0.07	0.23	0.49	0.84
Faraday efficiency [%]	99.6	98.4	98.0	97.5

For example, the experimental hydrogen production at 1.6 V was measured as 0.596 sccm at 500 °C, with a corresponding current density of 0.49 A cm^−2^. However, $\dot{V}$ for a SOEC operated at an electrolysis current density of 0.49 A cm^−2^ was calculated to be ≈0.608 sccm, which equaled a Faraday efficiency of 98.0% at this applied electrolysis voltage at 500 °C. As shown in Table 1, the steam electrolysis Faraday efficiency decreases from 99.6 to 97.5%, when the applied voltage increases from 1.2 to 1.8 V at 500 °C. The Faraday efficiency decrease at higher current densities may come from two reasons. One is the larger amount of heat generated from SOEC internal resistance than that required for water decomposition at high current densities because of increasing operating voltage.^36^ Another possible reason for the efficiency loss at higher current density may be the electronic or hole conduction, since the proton‐conducting oxides are not unity at high voltage.^37^ However, all the Faraday efficiencies at electrolysis voltages up to 1.8 V were close to the theoretical 100%, which indicated the current leakage through BZCYYb electrolyte could be negligible at 500 °C.

At durability test, the SOEC was operated at a constant electrolysis voltage of 1.6 V for 78 h. As shown in Figure 3d, the electrolysis current of SOEC with SAUP 3D steam electrode presented no degradation during the entire 78 h steam electrolysis process. Instead, the performance was constantly “activated” over the time since the current density increased steadily, consistent with the step test we observed (Figure 3c). However, SOEC with the conventional PBSCF steam electrode demonstrated obvious degradation at the same condition. The improved stability of SOEC with 3D electrode should be associated with the electrode microstructure rather than the material itself, since the only difference between these two cell configurations is the steam electrode structure. The EIS data for 3D PBSCF cell were acquired every 30 h and inserted in Figure S5 (Supporting Information). According to the EIS results, the cell total resistance presented a significant drop from 1.0 to 0.88 Ω cm^2^ within the initial 30 h and then gradually decreased to 0.85 Ω cm^2^ after 75 h. However, the polarization resistances of cell remain the same during the durability test, which was ≈0.15 Ω cm^2^. The Ohmic resistance, which accounted for more than 80% of the cell total resistance, decreased from 0.79 to 0.69 Ω cm^2^. Figure S6 (Supporting Information) provides a comparison of EIS results of SOEC with different steam electrode structure before and after durability test. Initially, the Ohmic resistances of SOEC with 3D and conventional PBSCF were very similar. The electrolysis performance difference was determined by the interfacial polarization resistance difference. After operation for ≈78 h, the Ohmic resistance of SOEC with conventional PBSCF increased gradually from 0.82 to 0.93 Ω cm^2^, while the interfacial polarization resistance remained at 0.7 Ω cm^2^. For SOEC with 3D steam electrode, the performance “activation” originated from the decrease of the cell Ohmic resistance. It is thus reasonable to attribute the electrolysis performance difference to the Ohmic resistance change. As a result, we hypothesize that the activation might be associated with the reorganization of steam electrode microstructure during the electrolysis. Our further SEM investigation provides solid evidence to support our hypothesis.

**Figure 4:**
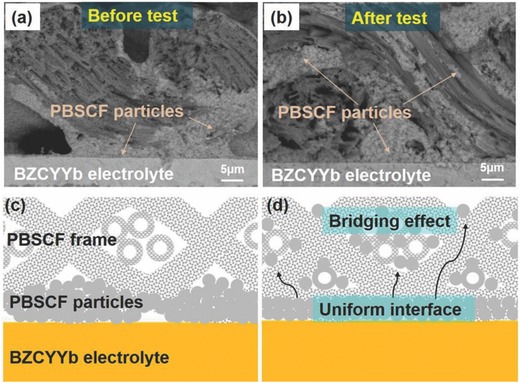
a,b) SEM image near steam electrode/electrolyte interface before and after steam electrolysis. PBSCF particles moved into the fiber crunches and made connections during the electrolysis process. Gaps between interface contacts disappeared due to the interface reorganization, which increase the active reaction area. c,d) Schematic diagrams of steam electrode/electrolyte interface before and after electrolysis present the interface reorganization “bridging” process due to the PBSCF particle migration. The gaps between interface contacts fade away due to the interface reorganization. As a result, the bridging process increased contacts between fibers and decrease the Ohmic resistance since more electron/proton pathways were generated.

**Figure** **4**a,b shows the cross‐sectional image near the interface between 3D steam electrode and BZCYYb electrolyte before and after steam electrolysis, respectively. As shown in Figure 4a, the PBSCF particles went through the electrode frame and mainly stack at the contact area between 3D electrode and electrolyte with the assistant of suspension liquid. Limited connection was observed between the fibers in the bulk frame. Obvious gap between interfacial contacts was also observed. After steam electrolysis, the fibers were connected with PBSCF particles and the previous PBSCF conjunction between frame and electrolyte became thinner. The gaps between interface contacts, demonstrated in Figure 4a and 2c, disappeared after electrolysis with a uniform electrode/electrolyte interface. The interface reorganization process was supposed to increase both the active reaction area and the electrolysis performance since the electrode/electrolyte interface is the key area to determine electrochemical cell performances.^38^ It should be noted that microcracks or spallation was also observed at the electrode/electrolyte interface, which may come from the SEM sample preparation since the 3D electrode was brittle after long‐term electrolysis. Figure 4c,d briefly demonstrated the mechanism of microstructure variation before and after electrolysis. Part of PBSCF particles moved from the interface to the gaps between electrode fibers and bridged them at the applied current and voltage during steam electrolysis. The improved contact between fibers could decrease the Ohmic resistance since more electron/proton pathways were generated. Combined with the EIS results in Figure 3d, the “bridging” effect between electrode fibers as well as the interface reorganization could be the reason for Ohmic resistance decrease and the performance activation.

A comparison of steam electrolysis performances (i.e., the electrolysis current densities at the applied voltage of 1.6 V) of typical P‐SOECs is shown in **Table** **2**, which clearly depicts how this work stands out from those reported in literatures. At present, almost all SOECs are operated between 600 and 900 °C. Our work showed significant progress in decreasing the electrolysis temperatures compared with those O‐SOECs that mainly operated above 750 °C. Among P‐SOECs, which is developed to operate at reduced temperatures, our work demonstrated advantages toward steam electrode performance which was reflected in the electrolysis current densities. For example, Bi et al. reported a durable P‐SOEC with current density of ≈100 mA cm^−2^ at 1.6 V, 600 °C.[[qv: 17a]] The current density at the same temperature in this work reached more than 2 A cm^−2^, which is almost 20 times of Bi's work. Gan et al. reported a short‐term stable proton‐conducting solid oxide steam electrolyzer with a current density of 1.3 A cm^−2^ at 1.6 V, 700 °C.^41^ However, the Faraday efficiency is only 22% and the long‐term durability test needs to be further evaluated.

**Table 2:** Comparison of performances of the state‐of‐the‐art SOEC technologies at different temperature ranges

Electrolyte	Steam electrode	Hydrogen electrode	*T* [^o^C]	Steam ratio	Applied voltage [V]	Current [A cm^−2^]	Ref.
BZCYYb	3D‐PBSCF	Ni‐BZCYYb	600 550 500	12%	1.3	0.85 0.42 0.21	This work
BZCYYb	Conventional PBSCF	Ni‐BZCYYb	600	12%	1.3	0.55	[12a]
BaCe_0.5_Zr_0.3_Y_0.2_O_3−_ *_δ_* (BCZY)	Sm_0.5_Sr_0.5_CoO_3_ ‐BZCY	Ni‐BZCY	600	50%	1.5	0.33	39
BZCYYb	NdBa_0.5_Sr_0.5_Co_1.5_Fe_0.5_O_5+_ *_δ_*	Ni‐BZCYYb	600	10%	1.3	0.75	19
BaCe_0.5_Zr_0.3_Y_0.16_Zn_0.04_O_3−_ *_δ_* (BZCYZ)	La_0.8_Sr_0.2_Mn_1−_ *_x_*Sc*_x_*O_3−_ *_δ_*	La_0.75_Sr_0.25_Cr_0.5_Mn_0.5_O_3−_ *_δ_* (LSCM)	700	5%	1.6	0.04	31
BCZYZ	Fe_2_O_3_‐LSM‐BCZYZ	LSCM‐BZCYZ	800	5%	1.6	0.07	40
BCZYZ	LSCM‐BCZYZ	Ni‐BZCYZ	700	3%	1.3	0.78	41
BaZr_0.9_Y_0.1_O_3−_ *_δ_* (BZY)	La_0.6_Sr_0.4_Co_0.2_Fe_0.8_O_3−_ *_δ_* (LSCF)‐BZY	Ni‐BZY	600	3%	1.3	0.05	[17a]

The steam electrolysis performance enhancement in our work is mainly attributed to the improvement of proton‐conducting electrolyte conductivity and the SAUP 3D framework that offers more pathways for steam molecules to reach the active reaction zone. The significant expansion of triple phase boundaries through sufficient contacts among steam, electrode, and electrolyte, which is proved to be the key factor influencing electrochemical cell performance,^42^ contributes to the performance enhancement. Further research will be conducted to investigate the influence of different steam ratios, novel steam electrode materials, and structures.

## 3 Conclusion

A high‐performance solid oxide electrolysis cell has been demonstrated by applying BaZr_0.1_Ce_0.7_Y_0.2−_ *_x_*Yb*_x_*O_3−_ *_δ_* as electrolyte, SAUP 3D PrBa_0.5_Sr_0.5_Co_2−_ *_x_*Fe*_x_*O_5+_ *_δ_* as steam electrode, and NiO‐BaZr_0.1_Ce_0.7_Y_0.2−_ *_x_*Yb*_x_*O_3−_ *_δ_* as hydrogen electrode. The electrochemical performance was tested at temperatures from 500 to 600 °C. The OCVs were close to the theoretical values at operating temperatures, indicating a good sealing and no gas leakage across the BZCYYb electrolyte. At 600 °C, the electrolysis current densities reaches −2.02 A cm^−2^ at 1.6 V. Constant activation was observed during long‐term electrolysis at applied voltage of 1.6 V at 500 °C. Our approach suggested a great prospective strategy of developing high‐performance SOECs at reduced temperature.

## 4 Experimental Section

**Figure 5:**
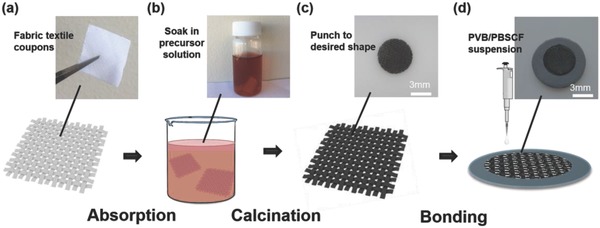
Process for fabricating SAUP 3D PBSCF textile steam electrode: a) initial fabric textile coupons in regular shape; b) fabric textile coupons soaked in precursor nitrate solution; c) PBSCF ceramic textile framework obtained after firing; d) bonding the 3D framework on electrolyte surface by filling with PVB/PBSCF suspension. Adsorption instead of absorption.

*Fabrication of SAUP 3D PBSCF Textile Steam Electrode*: The ceramic framework was fabricated through a template‐derived and self‐architectured procedure, which was first reported in a previous work.^43^ PBSCF precursor solution was prepared by dissolving a stoichiometric amount of Co(NO_3_)_2_·6H_2_O (Sigma Aldrich), Pr(NO_3_)_3_·6H_2_O (Sigma Aldrich), Fe(NO_3_)_3_·9H_2_O (Sigma Aldrich), Ba(NO_3_)_2_ (Sigma Aldrich), and Sr(NO_3_)_2_·6H_2_O (Sigma Aldrich) in distilled water. A fabric textile (Telio, Montreal, CA) was immersed in that precursor solution overnight, followed by firing at 750 °C for 4 h with a heating rate of 1 °C min^−1^ to form PBSCF ceramic textile. The microstructure of original fabric textile was characterized by SEM, shown in Figure S1 (Supporting Information). Coupons with a diameter of 3/16 in. were then punched from the sintered ceramic textile. The punched ceramic textile coupon was “sticked” on the top of a prepared anode supported NiO‐BZCYYb/BZCYYb half‐cell by using 10 wt% polyvinyl butyral (PVB)/PBSCF suspension (5 wt% PBSCF powders and 5 wt% PVB in ethanol) with a loading of 30 mL cm^−2^. After drying at 100 °C for 30 min, the cell with ceramic coupon was cofired at 750 °C for 2 h to form a full‐cell with ceramic textile steam electrode. The powder synthesis and half‐cell fabrication processes are described in the Methods Section, Supporting Information. The 3D steam electrode fabrication procedures are presented in **Figure** **5**.

*Electrolysis Cell Assembly and Testing*: The electrolysis button cell was then sealed in the house‐made reactor (Figure S2, Supporting Information) using glass sealant (Schott, Germany), with the steam electrode side up. Platinum mesh and wire were used as the current collector and leads, respectively. After assembly, the cell was then heated up to 600 °C with a ramping rate of 1 °C min^−1^. Oxygen (30 mL min^−1^) was used in steam electrode side at the ramping step while pure hydrogen, with a flow rate of 10 mL min^−1^, was fed into the hydrogen electrode side to reduce NiO into metallic Ni when the temperature reached 600 °C. After fully reduction, pure O_2_ was introduced into steam electrode after going through a bubbler at temperature of 50 °C. The steam partial pressure at steam electrode side was supposed to be 12% (50 °C) with a constant O_2_ flow rate of 100 mL min^−1^. In the hydrogen electrode, pure hydrogen was switched to 3% hydrogen in Ar as the sweeping gas. The steam electrolysis process started when a fixed current density was applied. The electrochemical performance was characterized and analyzed using a Solartron 1400 & 1470 electrochemical working station. The frequency range was between 0.1 and 1 × 10^5^ Hz with a 30 mV amplitude. Gas compositions at the hydrogen electrode side were analyzed using GC (Shimadzu 2010 plus) at open‐circuit voltage as well as at the different current densities to investigate the Faraday efficiency.

*Characterization*: The phase purity of BZCYYb powders and PBSCF textile was examined with a Rigaku SmartLab X‐Ray Diffraction in 15°–90° angular range with 0.04° step size and a 1.6 s resonance time. The patterns are shown in Figure S3 (Supporting Information). The textile anode microstructure, as well as cell cross‐sectional view, were both characterized via SEM (JEOL 6700F) with backscattering electron (BSE) analyzer and 3D X‐ray microscopy. TEM equipped with energy‐dispersive X‐ray spectroscopy (JEOL 4000 EX) was also used to investigate the element distribution of ceramic fibers.

## Conflict of Interest

The authors declare no conflict of interest.

## Supporting information

Supplementary
