# Supplementary material for: 3D Self‐Architectured Steam Electrode Enabled Efficient and Durable Hydrogen Production in a Proton‐Conducting Solid Oxide Electrolysis Cell at Temperatures Lower Than 600 °C
Source: Adv Sci (Weinh). 2018 Aug 31;5(11):1800360. doi: 10.1002/advs.201800360 (PMC6247067; doi:10.1002/advs.201800360)
Supplement: Supplementary file 1 — Supplementary [file ADVS-5-1800360-s001.pdf]

Copyright WILEY-VCH Verlag GmbH & Co. KGaA, 69469 Weinheim, Germany, 2018.

ADVANCED SCIENCE Open Access

Supporting Information

for *Adv. Sci.,* DOI: 10.1002/advs.201800360

3D Self-Architectured Steam Electrode Enabled Efficient and Durable Hydrogen Production in a Proton-Conducting Solid Oxide Electrolysis Cell at Temperatures Lower Than 600 °C

*Wei Wu, Hanping Ding, Yunya Zhang, Yong Ding, Prashant Katiyar, Prasun K. Majumdar, Ting He, and Dong Ding**

DOI: 10.1002/((please add manuscript number))
Article type: Full paper

Supporting Information

3D Self-Architectured Steam Electrode Enabled Efficient and Durable Hydrogen Production in A Proton Conducting Solid Oxide Electrolysis Cell at Temperatures Lower Than 600°C

*Wei Wu, Hanping Ding, Yunya Zhang, Yong Ding, Prashant Katiyar, Prasun K. Majumdar, Ting He, Dong Ding**

Methods

Powder Synthesis.

$BaZr_{0.1}Ce_{0.7}Y_{0.1}Yb_{0.1}O_{3-\delta}$ (BZCYYb) powder used for electrolyte and anode was prepared by the solid state reaction from stoichiometric precursors barium carbonate (Sigma Aldrich, ≥99%, $BaCO_3$), zirconium oxide (Alfa Aesar, 99%, ZrO_2), cerium(IV) oxide (Aldrich, 99.9%, CeO_2), yttrium(III) oxide (Alfa Aesar, 99.99%, Y_2O_3), and ytterbium(III) oxide (Alfa Aesar, 99.9%, Yb_2O_3). Powders were ball-milled for 24 h in ethanol, dried for 24 h, crushed, and calcined at 1100 °C for 10 h. The process was repeated to achieve the pure perovskite phase. The Cathode material $PrBa_{0.5}Sr_{0.5}Co_{1.5}Fe_{0.5}O_{5+\delta}$ (PBSCF) was synthesized by a glycine-nitrate process (GNP). Stoichiometric amounts of $Pr(NO_3)_3 \cdot 6H_2O$ (Alfa Aesar, 99.9%, metal basis), $Ba(NO_3)_2$ (Alfa Aesar, 99.95%), $Sr(NO_3)_2$ (Alfa Aesar, 99.97%), $Co(NO_3)_2 \cdot 6H_2O$ (Aldrich, 98+%) and $Fe(NO_3)3 \cdot 9H_2O$ (Alfa Aesar, 98+%) were dissolved in distilled water with proper amount of glycine. The solution was heated up to 350°C in air and followed by combustion to form fine powders which were calcined at 600°C for 4 h. The resulting powders were then grinded and calcined again at 900°C for 4 h.

Electrochemical Half Cell Fabrication.

Button cells with a configuration of NiO-BZCYYb|BZCYYb were fabricated by standard procedures. First, a mixture of NiO (Alfa Aesar) and BZCYYb (powder (weight ratio of 60:40) was mixed in ethanol and toluene using a high-energy ball mill (SPEX, 8000M) for 20 min. Plastizers and binders were added and then mixed for another 20 min to obtain a slip, which was de-gased and tape-casted to form green tapes. After drying overnight, the green tape was punched into wafers (12.7 mm in diameter), followed by pre-firing at 950°C for 2 h forming anode supports (~0.3 mm thick). Secondly, a thin layer of BZCYYb (~10 μm) was deposited on the anode support by a slurry coating process followed by co-firing at 1400°C for 4 h to form the final half cell.

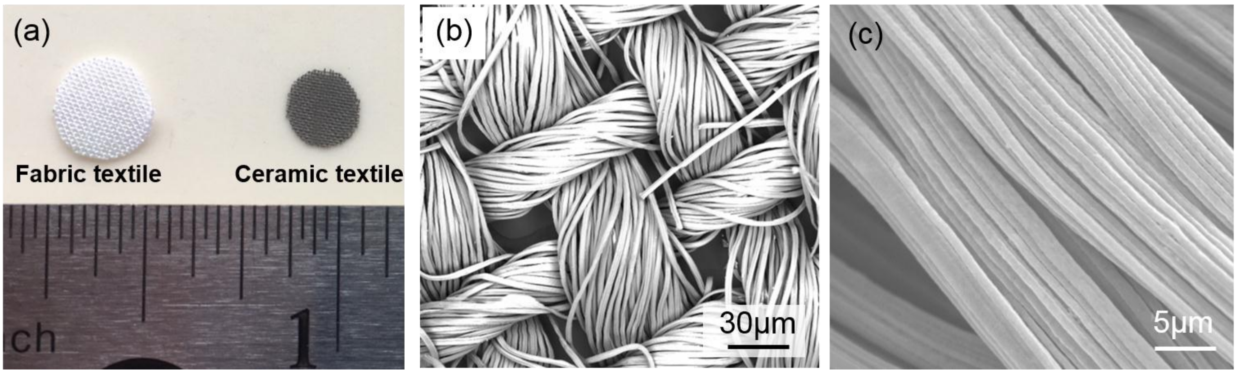

Figure S1. Characterization of fabric textile: (a) photography of fabric and ceramic textiles; (b) top view of fabric textile and (c) magnified image of solid fabric fibers. The self-architectured ceramic textile maintains the knit structure of original fabric textile.

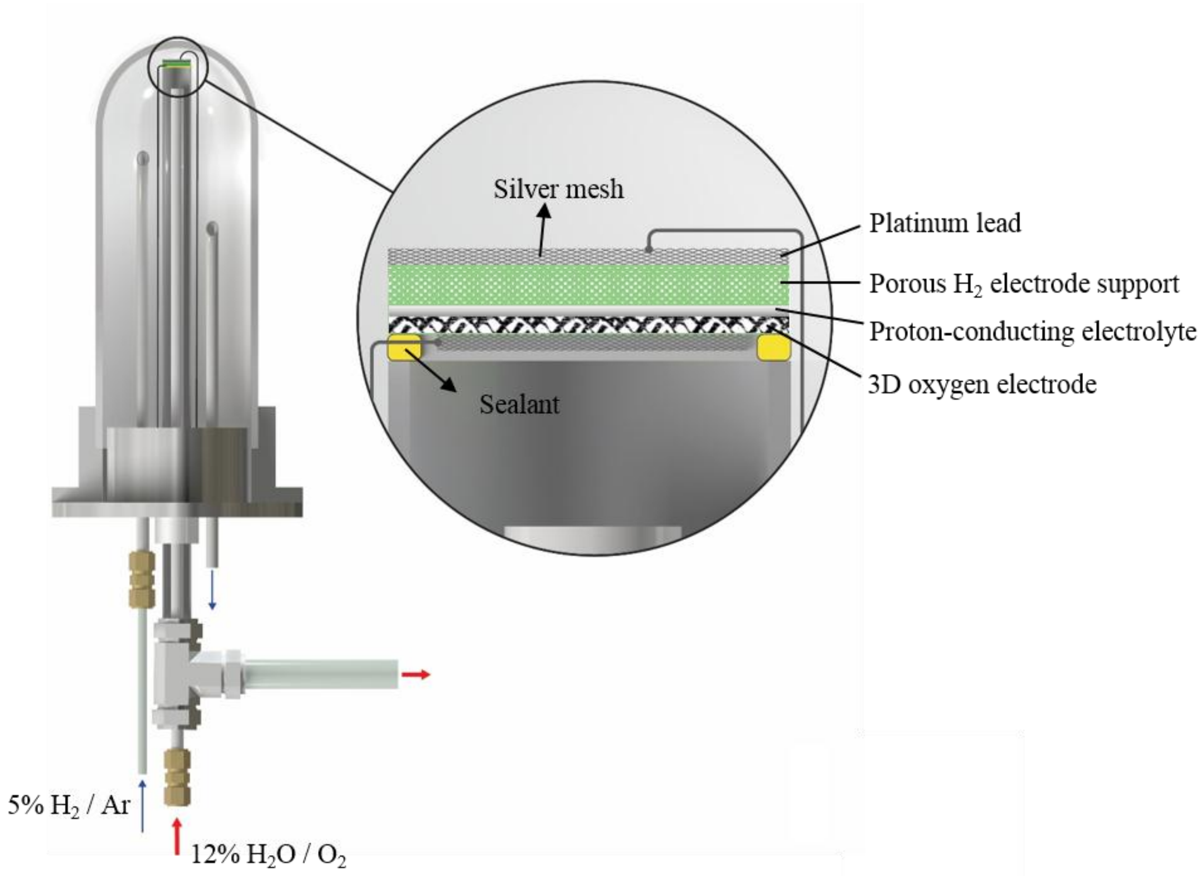

Figure S2. The schematic illustration of SOEC testing set up in this research.

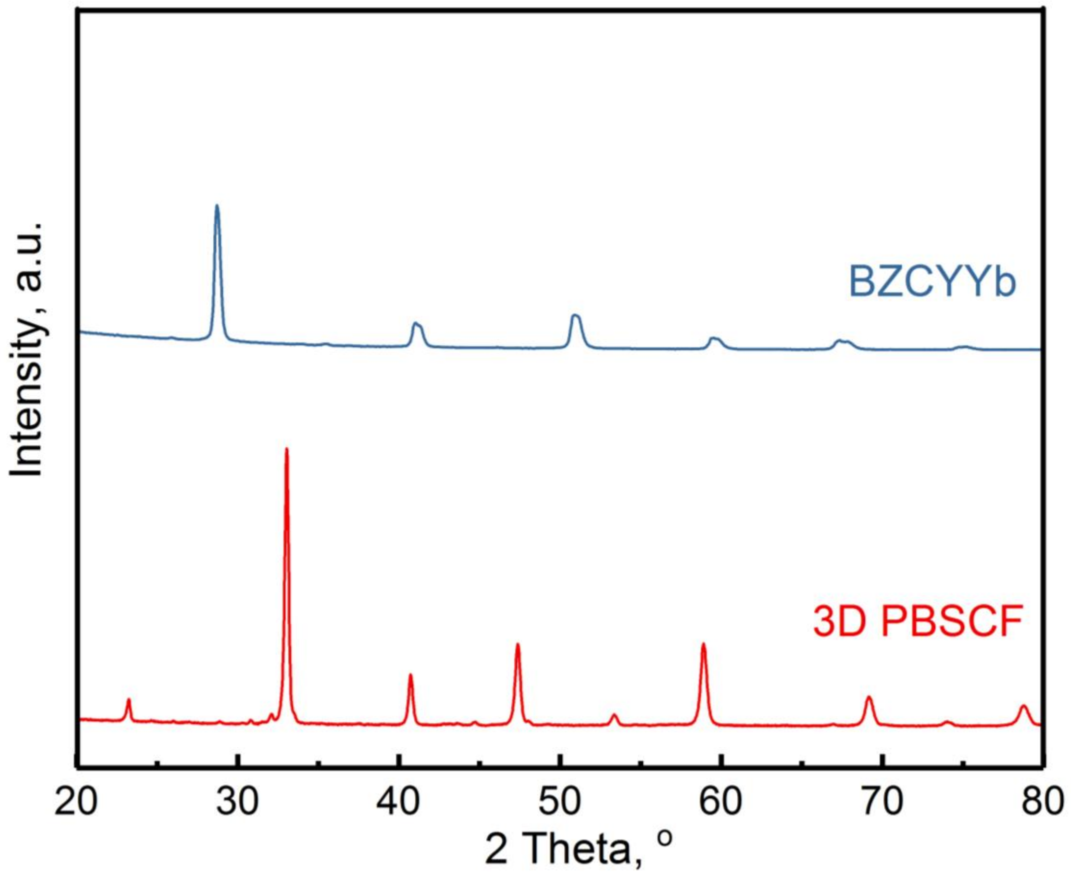

Figure S3. X-ray diffraction patterns for BZCYYb electrolyte sintered at 1400°C for 4 h and 3D PBSCF framework calcined at 750°C for 2 h. Perovskite BZCYYb and PBSCF phases are clearly identified and no secondary phases are detected.

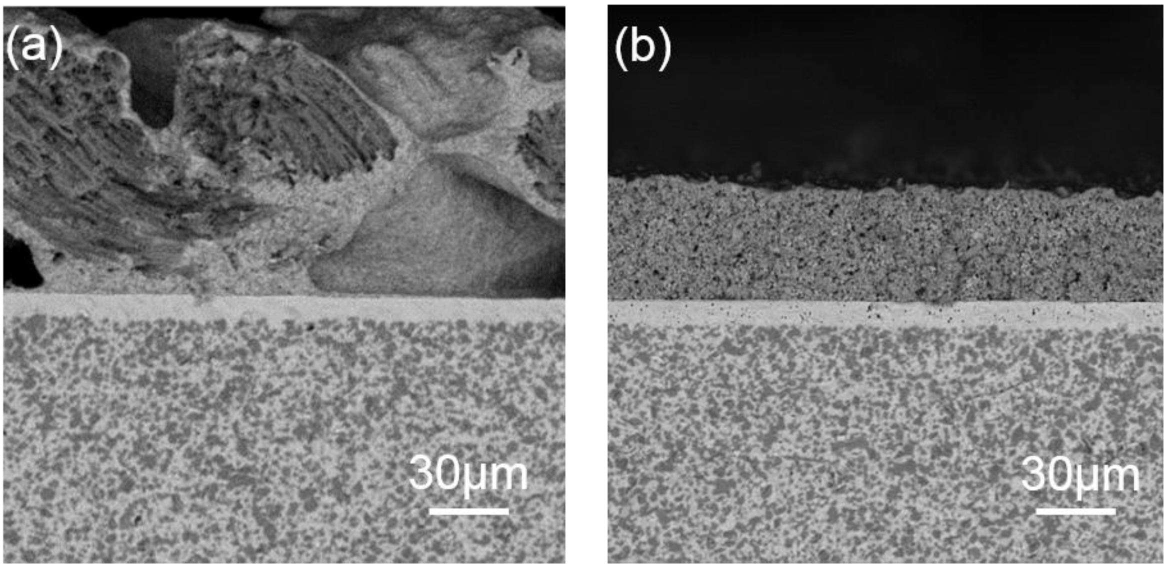

Figure S4. Cross-sectional images of as-prepared SOEC (a) with 3D PBSCF steam electrode and (b) conventional screen printed PBSCF steam electrode before steam electrolysis test. The 3D electrode exhibited remarkably difference in microstructure with the conventional electrode.

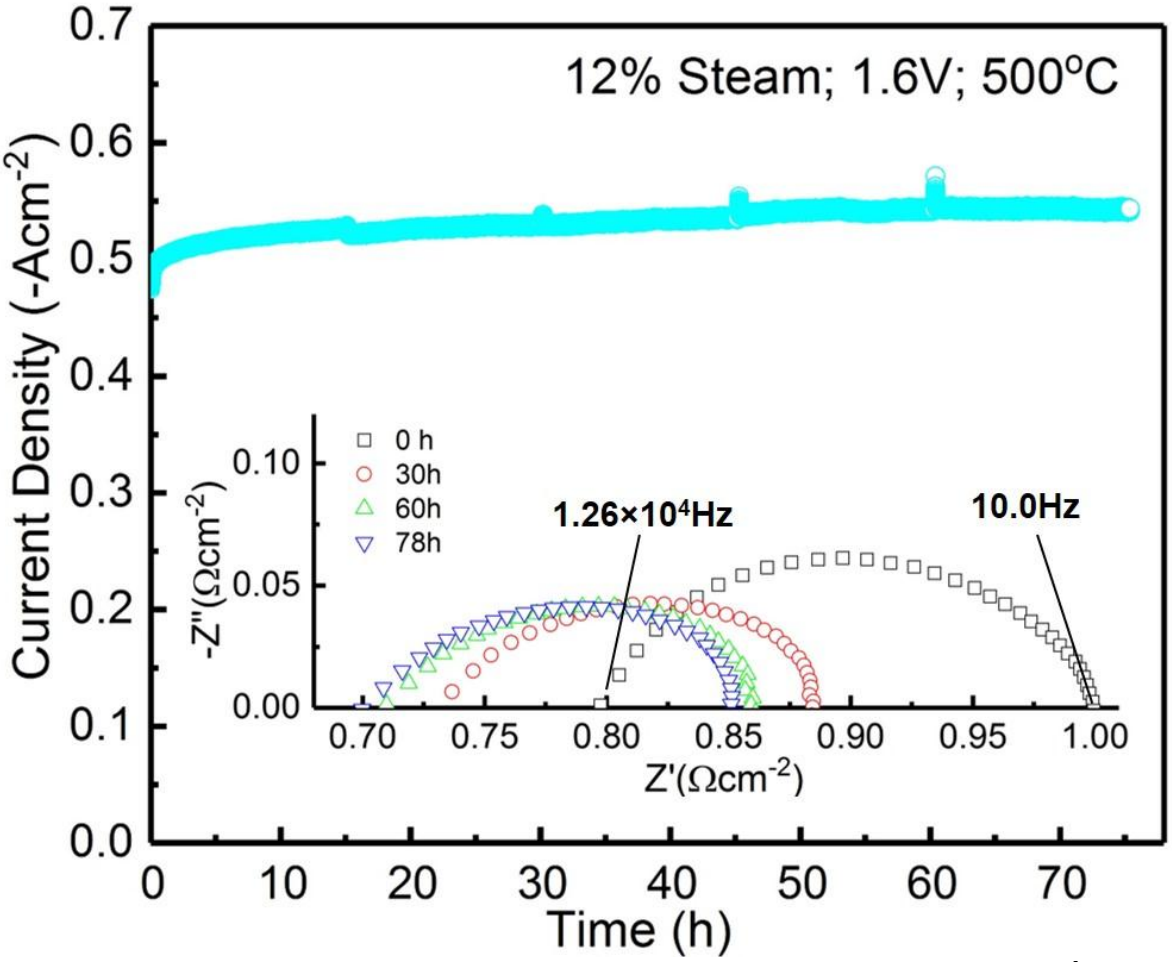

Figure S5. Current variation of 3D PBSCF SOEC at applied voltage of 1.6 V at 500°C, which demonstrates constant activation in electrolysis. EIS results at different period of time are inserted. H_2 and 12% H_2O–88% O_2 are used as the reacting gases in hydrogen electrode and steam electrode, respectively

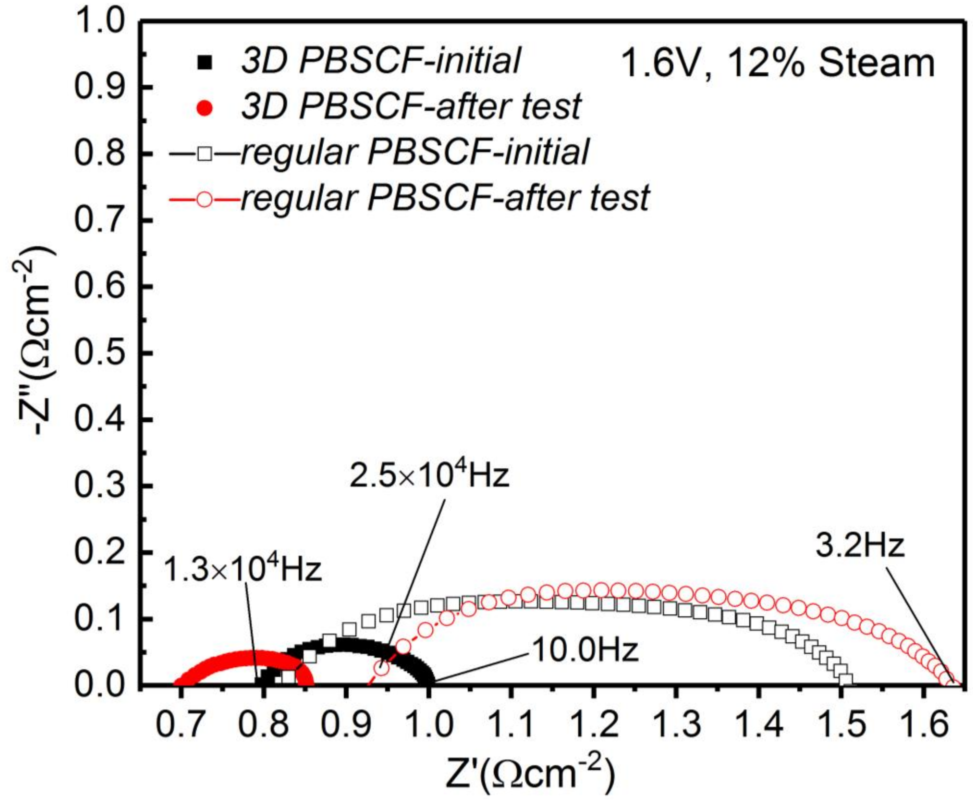

Figure S6. Electrochemical impedance spectra of cells with 3D PBSCF (solid) and conventional screen printed PBSCF steam electrode (hollow) before and after durability test under applied voltage of 1.6 V at 500°C. The decrease in polarization resistance of SOEC with 3D electrode determine the electrolysis performance enhancement. While the cell durability is mainly affected by the Ohmic resistance.
